# Management of EGFR-inhibitor associated rash: a retrospective study in 49 patients

**DOI:** 10.1186/2047-783X-17-4

**Published:** 2012-02-23

**Authors:** Peter Arne Gerber, Stephan Meller, Tatiana Eames, Bettina Alexandra Buhren, Holger Schrumpf, Sonja Hetzer, Laura Maximiliane Ehmann, Wilfried Budach, Edwin Bölke, Christiane Matuschek, Andreas Wollenberg, Bernhard Homey

**Affiliations:** 1 Department of Dermatology, University of Duesseldorf, Medical Faculty, Moorenstrasse 5, D-40225 Duesseldorf, Germany; 2 Department of Dermatology, Ludwig-Maximilians-University of Munich, Frauenlobstraße 9-11 & Thalkirchner Straße 48, 80539 Munich, Germany; 3 Department of Radiation Oncology, University of Duesseldorf, Medical Faculty, Moorenstrasse 5, D-40225 Duesseldorf, Germany

**Keywords:** EGFR, rash, papulopustular exanthema, erlotinib, cetuximab, panitumumab, gefitinib

## Abstract

**Background:**

In recent years inhibitors directed against the epidermal growth factor receptor (EGFR) have evolved as effective targeting cancer drugs. Characteristic papulopustular exanthemas, often described as acneiform rashes, are the most frequent adverse effect associated with this class of novel cancer drugs and develop in > 90% of patients. Notably, the rash may significantly compromise the patients' quality of life, thereby potentially leading to incompliance as well as dose reduction or even termination of the anti-EGFR therapy. Yet, an effective dermatologic management of cutaneous adverse effects can be achieved. Whereas various case reports, case series or expert opinions on the management of EGFR-inhibitor (EGFRI) induced rashes have been published, data on systematic management studies are sparse.

**Methods:**

Here, we present a retrospective, uncontrolled, comparative study in 49 patients on three established regimens for the management of EGFRI-associated rashes.

**Results:**

Strikingly, patients' rash severity improved significantly over three weeks of treatment with topical mometason furoate cream, topical prednicarbate cream plus nadifloxacin cream, as well as topical prednicarbate cream plus nadifloxacin cream plus systemic isotretinoin.

**Conclusions:**

In summary our results demonstrate that EGFRI-associated rashes can be effectively managed by specific dermatologic interventions. Whereas mild to moderate rashes should be treated with basic measures in combination with topical glucocorticosteroids or combined regiments using glucocorticosteroids and antiseptics/antibiotics, more severe or therapy-resistant rashes are likely to respond with the addition of systemic retinoids.

## Background

In recent years inhibitors directed against the epidermal growth factor receptor (EGFR) have evolved as effective cancer-targeting drugs [1]. These drugs include monoclonal anti-EGFR antibodies, such as cetuximab or panitumumab, as well as small molecule EGFR tyrosine kinase inhibitors, such as erlotinib or gefitinib. Additionally, current studies report promising results on the clinical effectiveness of drugs that target the EGFR-signaling cascade, such as the BRAF inhibitor vemurafenib or MEK inhibitors [2]. Characteristic inflammatory papulopustular exanthemas, often described as acneiform or rosaceaform rashes, are the most frequent adverse effect associated with the use of EGFR-inhibtors (EGFRI) [3-6]. Within the first days to weeks of therapy > 90% of patients develop these rashes. In the majority of cases skin lesions initially appear within areas of skin that bear high densities of seborrheic glands. However, the rash may progress into other areas, generalize in the course, or progress into perifollicular xanthoma [7]. Notably, recent studies have demonstrated that rash appearance and severity are correlated positively with the anti-tumor effect of the EGFRI [8,9]. Accordingly, the rash is regarded the best surrogate marker for clinical response to EGFR-targeting drugs [9]. Besides the rash, patients may develop additional dermatologic adverse effects, including pruritus, paronychias, infections, or impressive alterations of eyebrows and lashes [5,6,10-16]. Another notable aspect of EGFRI-associated cutaneous adverse effects is the severe radiation dermatitis following additional radiation therapy [17-20]. However, radio therapy prior to initiation of EGFRI therapy may also prevent rash development [21].

Taking into account the broad spectrum and the potential severity of EGFRI-associated adverse effects, it is reasonable that these toxicities may significantly compromise the patients' quality of life (QoL), thereby potentially leading to incompliance as well as dose reduction or even termination of the anti-EGFR therapy. Hence, effective management regimens are urgently needed.

Here, we report the results of a retrospective study designed to compare the effectiveness of established rash management strategies in EGFRI-associated rash development.

In our study patients were treated using one of three rash-management strategies: (1) sole topical anti-inflammatory measures (mometason furoate cream); (2) combined topical anti-inflammatory (prednicarbate cream) and anti-infectious measures (nadifloxacin cream); and (3) combined topical anti-inflammatory (prednicarbate cream), anti-infectious measures (nadifloxacin cream) as well as concomitant systemic isotretinoin therapy. All have previously been reported to be effective by several independent case reports and guidelines [5,10,22-25]. After three weeks of treatment, patient rashes were re-assessed to determine the effectiveness of each strategy.

## Methods

### Assessment of rash severity

**Figure 1:**
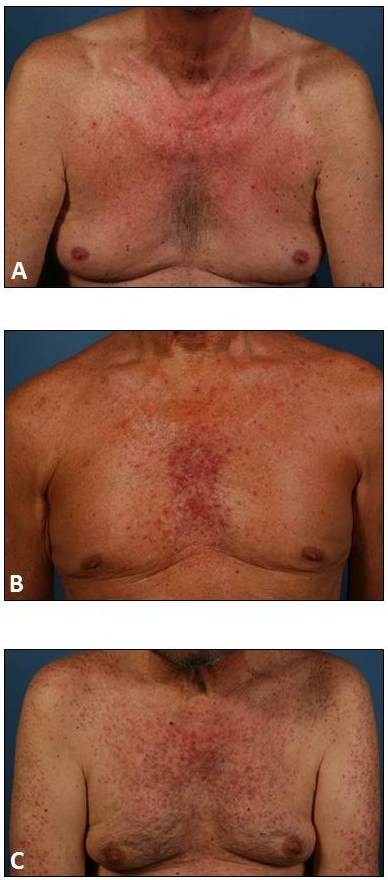
**Severity of EGFRI-induced papulopustular rashes**. Rash severity was assessed using the EGFRI-induced rash severity score (ERSS). ERSSs may range from 0 (no skin affection), over (**A**) 1 to 20 (mild), (**B**) 20 to 40 (moderate), up to (**C**) scores exceeding 40 points, indicating severe cases.

Rash severity was assessed during the initial presentation to our clinics (Departments of Dermatology, University Hospital Düsseldorf and Ludwig-Maximilian-University of Munich) and after three weeks of specific dermatologic therapy. Rash severity was assessed applying the EGFRI-induced rash severity score (ERSS or WoMoScore), a skin-specific scoring system introduced in 2008 [26]. Briefly, the ERSS is a combined score of the severity of five different aspects of the EGFRI-rash (color of erythema, distribution of erythema, papulation, pustulation and scaling/crusts), combined with a score based on the extent of affected facial area and the total body area involved. ERSSs range from 0 (no skin affection), 1 to 20 (mild), between 20 and 40 (moderate), up to scores exceeding 40 points, indicating severe cases (Figure 1) [26].

### Patient selection criteria

Selection criteria included patients treated with cetuximab or erlotinib that suffered from EGFRI-associated rash at the time of referral. The selection was limited to initial patients and their follow-up visits in the time frame of March 2007 to October 2009. We enrolled 49 patients who presented with an ERSS of 10 or higher. The study was approved by the local ethics committees.

### Treatment

In stage 1 of the study, 21 patients (ERSS 10.3 to 77.9) were treated topically with mometason furoate cream twice daily for three weeks. In stage 2 of the study, 23 patients (ERSS 12.5 to 67.1) were treated topically with nadifloxacin 1% cream once daily in the morning in combination with prednicarbate 0.25% cream once daily in the evening for three weeks as described [22,27]. In stage 3 of the study, five patients with an ERSS > 50 received topical nadifloxacin 1% and prednicarbate 0.25% cream in combination with the systemic retinoid isotretinoin 10 to 20 mg/d for three weeks as described [25]. Adverse effects of our management strategies were generally rare and in line with the potential common adverse effects reported for each drug in the literature.

### Statistical analysis

Statistical analysis was performed using the Student's t-test.

## Results

**Figure 2:**
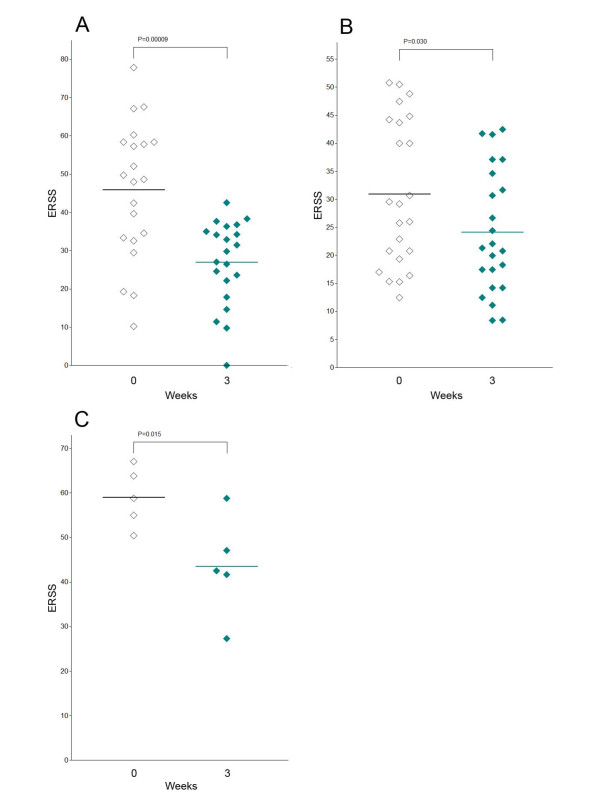
**Significant improvement of rash severity under specific dermatologic measures**. (**A**) Topical mometason furoate cream significantly (*P *= 0.0009) improved the severity of the skin rash (ERSS) in patients treated with EGFRI after three weeks. (**B**) A combined topical regimen with prednicarbate cream and nadifloxacin cream significantly (*P *= 0.03) improved the ERSS in patients treated with EGFRI after three weeks. (**C**) A triple therapy with topical prednicarbate cream, topical nadifloxacin cream and systemic isotretinoin significantly (*P *= 0.015) improved the ERSS in patients treated with EGFRI after three weeks. Statistical analyses were performed applying the Student's t-test.

In this study we sought to compare the effectiveness of established rash management strategies. Therefore, we first assessed the efficacy of a potent anti-inflammatory topical glucocorticosteroid with low-atrophogenic potential [28]. Twenty-one patients (ERSS ranging from 10.3 to 77.9) were treated with mometason furoate cream. Assessment of the ERSS prior to therapy initiation and after three weeks revealed that the mean rash severity improved significantly (*P *= 0.00009) from 45.9 to 27.0 and demonstrated the efficacy of our approach (Figure 2A).

The second regimen used, a combined approach, which targets the inflammatory as well as the infectious facet of the rash. Twenty-three patients (ERSS ranging from 12.5 to 67.1) were treated with nadifloxacin 1% cream, a potent topical fluoroquinolone antibiotic with a broad-spectrum activity against gram-positive and gram-negative bacteria, as well as the topical glucocorticosteroid prednicarbate 0.25% cream as described previously [24]. Assessment of the ERSS revealed that the mean rash severity improved significantly (*P *= 0.03) from 30.9 to 24.8 after three weeks, demonstrating the efficacy of our approach (Figure 2B).

Finally, we included the retinoid isotretinoin that represents a standard option for the treatment of papulo-pustular skin diseases like acne or rosacea [4,29]. Moreover, isotretinoin has been reported to be effective in the management EGFR-antagonist rashes [5,25]. Five patients, which presented with severe ERSS of > 50 or therapy-resistant courses were treated with nadifloxacin 1% cream, prednicarbate 0.25% cream, and systemic isotretinoin (10 to 20 mg/day) [5,25]. Interestingly, these severely affected patients significantly improved during isotretinoin treatment (*P *= 0.015) and demonstrated on average a reduction of the ERSS from 59.2 to 43.8 after three weeks of therapy (Figure 2C).

All results are summarized in Table 1.

**Table 1:** Significant improvement of rash severity under specific dermatologic measures

Therapy	Patients (*n*)	ERSS (mean)		ERSS (mean)	Significance (*P*)
		**Day 0**	**Day 21**	**Reduction**	

Mometason furoate cream (2x/d)	21	45.9	27.0	18.9	0.00009
Nadifloxacin 1% cream (1x/d) plus prednicarbate 0.25% cream (1x/d)	23	30.9	24.8	6.1	0.03
Nadifloxacin 1% cream (1x/d) plus prednicarbate 0.25% cream (1x/d) plus systemic isotretinoin (10-20 mg/d)	5	59.2	43.8	15.4	0.015

Patients with EGFRI-induced rash were treated with three different therapy regimens. All therapies resulted in a significant (*P *< 0.3) improvement of the severity of the skin rash (ERSS) after three weeks. Statistical analyses were performed applying the Student's t-test.

## Discussion

Today, there is a broad variety of independent case reports and guidelines on different options for the management of EGFRI-associated rashes [5,22-25]. Yet, studies that compare different therapeutic regimens and analyses in larger collectives of patients are sparse. Accordingly, we conducted a comparative analysis of the clinical efficacy of different EGFRI rash management strategies that target the inflammatory and/or the infectious characteristics of the rash. Notably, our results demonstrate that all approaches were effective and significantly reduced the severity of the rash over a period of three weeks.

The statistically most significant effects were achieved with topical mometason furoate cream (*P *= 0.00009), followed by topical prednicarbate cream plus nadifloxacin cream plus systemic isotretinoin (*P *= 0.015) and finally topical prednicarbate cream plus nadifloxacin cream (*P *= 0.03). However, statistical comparison of different therapy regimen is limited due to variations in patient numbers and rash severity in each of the three test groups before therapy. Topical mometason furoate achieved the highest mean ERSS-reduction with 18.9 points, followed by topical prednicarbate cream plus nadifloxacin cream plus systemic isotretinoin with 15.4 points and topical prednicarbate cream plus nadifloxacin cream with 6.1 points. Moreover, topical mometason furoate was the only therapy that resulted in a complete resolution of all rash symptoms in one patient. Yet, it must be noted that statistical significance is highly dependent on the number of patients included in each group, and because the ERSS system was designed with a non-linear affected-area scale emphasizing minor variations in mild patients with face involvement only [26].

Mometason furoate alone appeared to be more effective than prednicarbate plus topical nadifloxacin. However, mometason furoate is the more potent glucocorticosteroid (class III) as compared to prednicarbate (class II) and therefore represents a higher risk of inducing steroid-associated adverse effects, such as skin atrophy [30]. Nevertheless, it is questionable, whether these adverse effects may play a role in the short-term treatment of EGFRI rashes, as inflammatory skin lesions have been shown to slowly regress even without therapy in the course of sustained EGFRI-therapy. Topical nadifloxacin was administered to target the infectious component of the rash [10]. Future studies may analyse the efficacy of a combination of topical momentason furoate plus nadifloxacin.

With regard to the variation in significance and over-all efficacy of the different approaches, it must be noted that we compared three somewhat heterogenous patient groups. Whereas patients with varying ERSS were randomly subjected to therapies with topical mometason furoate or topical prednicarbate cream plus nadifloxacin cream, the addition of systemic isotretinoin was limited to patients that were severely affected and presented either with a very high ERSS (> 50) or patients that were referred to our clinics due to rashes that were therapy-resistant to other approaches (such as topical antibiotics or topical glucocorticosteroids). Accordingly, effects observed for systemic isotretinoin may not have been as dramatic when compared to sole topical prednicarbate plus topical nadifloxacin or topical mometason furoate.

With regard to study design, it may be criticized that we did not compare the tested conditions to negative controls, such as a subgroup of EGFRI patients whose rash was left untreated for the study period. Yet, an untreated or insufficiently managed rash can significantly compromise the patients' QoL and patients included in our analysis had initially been referred to us specifically for the treatment of their cutaneous adverse effects by their treating oncologists.

Notably, all approaches that were analysed in this study are in line with recent expert recommendations that suggest an escalating strategy for the management of the EGFRI rash [5,6,16] with a succession of treatments, as indicated, summarized as follows: intensive skin care in combination with mild cleansers, followed by the use of mild (class II) to moderate (class III) potent topical glucocorticosteroids with low atrophogenic potential such as hydrocortisone butyrate, prednicarbate (both class II), methylprednisolone aceponate or momethason furoate (both class III). In fact, our results demonstrate a significant efficacy of topical glucocorticosteroid monotherapy. Taking into account the high incidence of bacterial superinfections of the EGFRI rash, alternative recommendations include the combination of mild topical glucocorticosteroids and topical antibiotics or antiseptics with low cytotoxic potential [31]. Recent studies report infections at the sites of dermatologic adverse effects in 38% of EGFRI rash patients. A detailed microbiologic analysis of these cutaneous infections identified *Staphylococcus aureus *in 59.5% of the cases [10,32]. Nadifloxacin is a potent topical fluoroquinolone antibiotic hence representing a probable candidate to target superinfections in EGFRI rash patients. In fact, we could show that the combination of nadifloxacin 1% cream and prednicarbate 0.25% cream significantly improved rash severity. In this context the management of cutaneous infections is also likely to exert protective effects regarding the aggravation of skin inflammation as infectious agents may trigger inflammatory rash progression by means of "Koebnerization" [33]. Systemic isotretinoin, finally, is recommended for the management of severe EGFRI rashes of rashes that do not respond to other therapies [23]. Hence, in our study, patients with an ERSS > 50 were subjected to a combined management approach with nadifloxacin 1% cream and prednicarbate 0.25% cream as well as systemic isotretinoin [25]. Our results demonstrate that even severe rashes can be improved significantly by this approach. Yet, is must be noted that the use of systemic isotretinoin in EGFRI patients is controversial, since potential antagonism of the anti-tumor effect of the EGFRI is possible, although this has not been investigated systematically yet. Nevertheless, similar arguments may be proposed for any systemic approach, such as the administration of oral tetracyclines as rash prophylaxis [34,35].

## Conclusions

In summary our results demonstrate that EGFRI-associated rashes can be effectively managed by specific dermatologic interventions. Whereas mild to moderate rashes should be treated with basic measures in combination with topical glucocorticosteroids or combined regiments using glucocorticosteroids and antiseptics/antibiotics, more severe or therapy-resistant rashes are likely to respond with the addition of systemic retinoids. Additional options include systemic antibiotics or systemic glucocorticosteroids. Finally, novel approaches have been proposed to abrogate EGFR-inhibition specifically in the skin. One such option is the ligand-independent activation of the EGFR by topical application of vitamin K analogues, such as vitamin K1 or vitamin K3 (menadione) [36-39]. Yet, additional systematic studies are urgently needed to quantify and compare the effectiveness and adverse effects of EGFRI rash-management strategies.

## Competing interests

The authors declare that they have no competing interests.

## Authors' contributions

PAG, SM, and BH participated in the design of the study and performed the statistical analysis. PAG, BH, EB, SM, TE, BAB, HS, SH, LME, AW, CM and WB helped to design and draft the manuscript. All authors read and approved the final manuscript
